# Interface Reactions and Synthetic Reaction of Composite Systems

**DOI:** 10.3390/ma3010264

**Published:** 2010-01-08

**Authors:** Joon Sik Park, Jeong Min Kim

**Affiliations:** Division of Advanced Engineering Materials, Hanbat National University, Daejeon, 305-719 Korea; E-Mail: jmk7475@hanbat.ac.kr (J.M.K.)

**Keywords:** reactive diffusion, biased reaction, chemical potential

## Abstract

Interface reactions in composite systems often determine their overall properties, since product phases usually formed at interfaces during composite fabrication processing make up a large portion of the composites. Since most composite materials represent a ternary or higher order materials system, many studies have focused on analyses of diffusion phenomena and kinetics in multicomponent systems. However, the understanding of the kinetic behavior increases the complexity, since the kinetics of each component during interdiffusion reactions need to be defined for interpreting composite behaviors. From this standpoint, it is important to clarify the interface reactions for producing compatible interfaces with desired product phases. A thermodynamic evaluation such as a chemical potential of involving components can provide an understanding of the diffusion reactions, which govern diffusion pathways and product phase formation. A strategic approach for designing compatible interfaces is discussed in terms of chemical potential diagrams and interface morphology, with some material examples.

## 1. Introduction

Interdiffusion reactions often occur for two phase reinforced metal matrix composite systems, and the limited availability of components restricts potential combinations and successful compatibility. The constraints often lead to diffusion barrier coatings, when selected materials possess superior properties. While the diffusion barrier coating strategy can be effective, it adds complexity to the overall processing and can represent a weak point in the system; especially when the large amount of interfacial area that must be covered without any flaws is considered [1,2,3]. Required to serve multifunctional purposes, a composite is usually a complex system of multiphase materials. In a composite with a 30% volume fraction fibers with a diameter of 10 μm the matrix and reinforcement interfacial areas can approach 10^3^ cm^2^/cm^3^ so that a large amount of interfacial area exists for reactions [4], and consequently composites behave as an interfacial material, because their processing and performance are dominated by the characteristics of internal interfaces [1,2,3,4,5,6,7]. From this perspective, an *in situ* processing approach is to design the composite members for intentional reactions to yield a final state of a thermodynamically compatible system in which both an initial matrix and reinforcing materials undergo compositional and structural changes to yield a final combination, at least for the local interface area [4]. 

When composite members experience compositional changes, intermediate phases are usually produced, and the formation of these intermediate phases is dictated by a consideration of multi-component phase equilibria and diffusion pathways [8]. The properties of the produced intermediate phases provide a critical factor for determining the composite’s properties. In this regard, the reliable prediction of the diffusion path, including initial experimental data, is necessary to achieve *in situ* an optimized composite. Furthermore, the accumulated data and an effective strategy for controlling interface reactions can extend useful fields such as packaging and/or coating process. Several studies have concentrated on the analysis of diffusion phenomena in multi-component systems [9]. While basic diffusion analyses have been developed, an effective strategy to control the diffusion pathway has not been well-developed. If the reaction between matrix and reinforcement materials does not yield a preferred phase, the unwanted diffusion path should be changed to yield a desired phase sequence. In reality, this objective must be achieved by analyzing the interface reactions. While controlling the diffusion pathway is attractive, the designed diffusion pathway may not be stable, so that it is worthwhile to investigate the thermodynamic stability and kinetics of the product phases. 

SiC is one attractive material with superior properties such as corrosion resistance, high strength and metallization [10,11,12]. A number of studies on the SiC-Metal interface reactions have been reported and include investigations on the thermal compatibility and/or the solid state reactions [13,14,15,16,17,18]. The reaction product sequence and interface morphology in SiC/metal reactions depend mainly on the contact materials. The products of an SiC/Ni reaction, for example, show a periodic morphology composed of alternating carbon and silicides [19,20]; in contrast, the products of the an SiC/Cr reaction reveal planar layers of carbides and silicides [21]. For the SiC/metal reactions, the irregular morphology of product phases with complex kinetics during interdiffusion reactions has caused a difficulties in understanding the kinetic behavior of SiC/metal. Even though the estimation of kinetic values is well treated, the diffusion coefficients necessary for complete analyses are generally unavailable. Furthermore, they require four diffusion coefficients for each phase and they are variable with composition at a planar shape interface [9]. 

The restriction requires a semi-empirical estimation that may be generally applicable to useful systems, which does not limit a complex calculation nor does present phenomenological explanations. In fact, since the produced phase during interdiffusion reactions (diffusion pathway) can determine the global property of materials such as composites, multilayers or coatings, the clear identification of developed phases is crucial for optimization of materials’ properties. In this perspective, chemical potentials of involving components may provide an insight for understanding the diffusion pathways during interdiffusion reactions. 

For the current review, a graphical methodology for constructing chemical potential diagrams of ternary systems is presented for components with a limited solubility. At the same time, an engineering concept for achieving stable interfaces in a kinetic bound is discussed in terms of additional layer selection by controlling diffusion pathways under the examination of component chemical potentials for selected Ti-Al-Si and SiC/metal systems. 

Intermetallic compounds such as the TiAl and Ti_3_Al phases in the binary Ti-Al system have shown favorable properties and provide a potential to increase their toughness by an addition of other elements. They have been studied for the replacement of high temperature materials by producing composites composed of soft or hard reinforcement materials [22]. The Ti-Si system is another candidate for high temperature structural materials, because several high temperature intermetallics are present in the system. Moreover, the system includes the Ti_5_Si_3_ phase as a potential reinforcement material, because its melting temperature is 2,130 °C and the compression strength (0.2% offset strength) has been reported up to 1,050 MPa at 1,273 K [23,24]. Therefore, the ternary Ti-Si-Al system offers potentially useful properties for high temperature applications. 

In this review, special attention has been given to analysis and control of the interface reactions and to develop strategies for the selected ternary model systems such as Si-C-Metal and/or Ti-Al-Si system. The identification and controlling reaction pathways have been examined by concentrating on the phase evolution, and kinetic and thermodynamic stability in terms of the relevant chemical potential variations. 

## 2. Chemical Potential Diagrams of Ternary Systems

Since the diffusion path and phase sequence can be affected by the chemical potential changes during a reaction, it is important to analyze the chemical potential variation of each component. One method to understand a diffusion path is based upon the stability diagram concept proposed by van Loo [25]. Generally, the stability diagram represents an activity variation of a component as a function of composition. In order to develop the methodology, it is necessary to identify a complete routine to estimate chemical potential values. 

The basic requirement for constructing the chemical potential diagram is to estimate the free energy values of all possible phases based upon the equivalent reference states so that the free energy values of intermediate phases can be compared with respect to the same reference state. Then, the stable free energy trajectory is projected on the ternary isothermal phase diagram (the free energy plane is represented by a curved surface in a ternary system). The three chemical potential values at a specified composition (at the point M (X_A_, X _B_, X_C_), where X_A_+X_B_+X_C_=1 and X_i_ is the mole fraction of i) for a ternary solution may be expressed as equation (1)-(3) [26]:

µ_A_ = G_m_ – X_B_(∂G_m_/∂X_B_) – X_C_(∂G_m_/∂X_C_) (1)

µ_B_ = G_m_ – (1-X_B_)(∂G_m_/∂X_B_) – X_C_(∂G_m_/∂X_C_) (2)

µ_C_ = G_m_ – X_B_(∂G_m_/∂X_B_) – (1-X_C_)(∂G_m_/∂X_C_) (3)

where µ_i_: chemical potential of i component, G_m_: molar Gibbs free energy at a point and X_i_: mole fraction of component i. 

Graphically, the three intercept points between the designated tangential plane and the vertical axis of each of the three pure elements indicate the chemical potential values at a certain composition (see [26] for further detailed analyses). For systems with a large solubility the chemical potential (activity) values of components with respect to the composition changes are generally obtained based upon the availability of the free energy values. While for many ternary systems the free energy data with respect to large composition changes is lacking, the free energy values of compounds that have a limited solubility are relatively well-determined. Actually, for the systems that have a limited solubility (assumed to be a linear compound), it is only required to have free energy values of those compounds at a given composition, assuming that they are linear compounds, in order to calculate the chemical potential values. 

**Figure 1:**
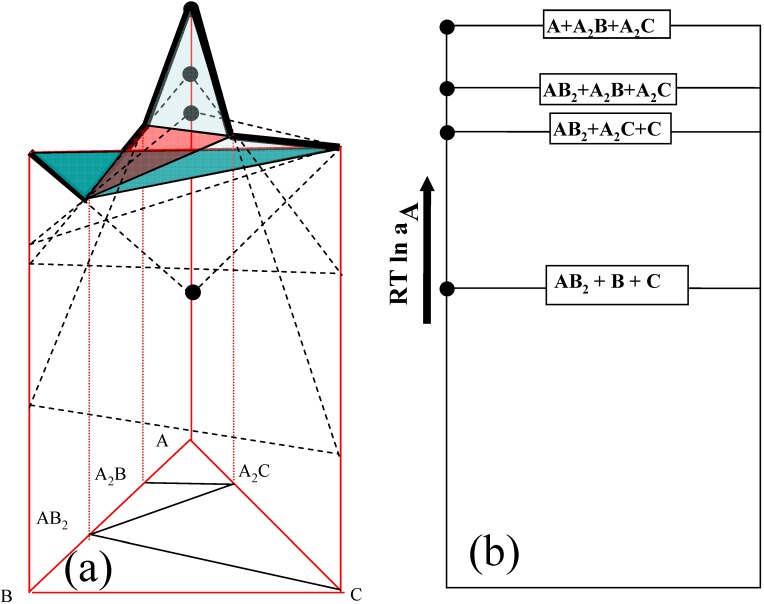
Schematic diagram of (a) three-dimensional tangential plane of free energy and (b) two- dimensional chemical potential diagram [6].

**Figure 2:**
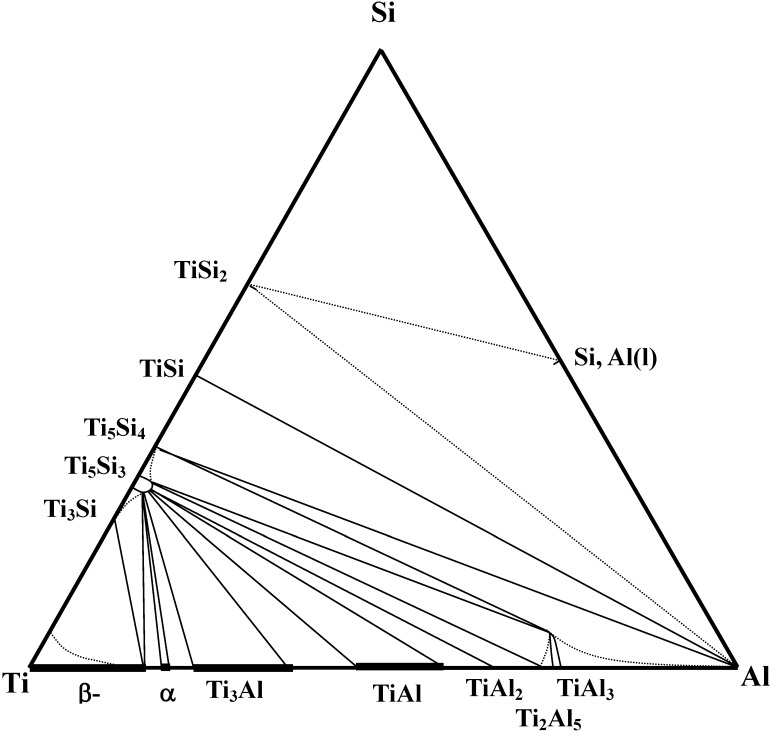
Calculated isothermal phase diagram of the Ti-Si-Al system at 1,373 K [7].

The tangential chemical potential planes of three components in a hypothetical A-B-C ternary system are constructed with respect to a simplified model isothermal ternary diagram in Figure 1, in which only four three-phase equilibrium regions exist; A -A_2_B- A_2_C, A_2_B-A_2_C- AB_2_, AB_2_-A_2_C-C and AB_2_-B-C. Based upon the tangential planes of Figure 1(a) the chemical potential diagram of component A is constructed in Figure 1(b). It should be noted that the tangential free energy value of a three phase field is expressed as a line, and that of a two phase field is placed between three phase regions (between lines) in Figure 2b. For example, for the A_2_B-A_2_C- AB_2_ three phase equilibrium region, the chemical potential value of component A may be determined as *µ_A_*(*at* *A*_2_*B* = *µ_A_*(*at* *AB*_2_) = *RT*ln*a_A_*, where *a_A_*: activity of component A . It should be mentioned that the chemical potential value of component A is determined by the two phase equilibrium of A_2_B - AB_2_ in this model ternary system. In the similar routine, the chemical potential values of other equilibrium regions can be determined for all of the equilibrium planes as shown in Figure 1(b). Again, if the same graphical method is applied to find chemical potential values, the values of µ_B_ and µ_C_ can be obtained. The virtue of this analysis is that the chemical potential (and/or activity) of a component on each three phase equilibrium plane can be obtained with a relatively simple calculation by using a graphical method, and thus the chemical potential changes can be identified upon developed intermediate phases (diffusion pathway) during interdiffusion reactions. 

## 3. Thermodynamic Stability of Ti-Si-Al and SiC/Me Reactions

Since Al in the Ti-Si-Al system has a low melting temperature compared to Ti and Si, previous studies have concentrated on the equilibria in the Ti-rich area. After the partial isothermal sections were reported by Crossley and Turner [27], several studies have been published, including five ternary phases [28,29,30,31,32]. However, since incomplete binary phase diagrams of Ti-Al system and Ti-Si system have been used to identify phase equilibria, the published isothermal sections do not indicate the binary phase equilibria properly. For example, the isothermal sections at 823 K and 1,473 K [28] do not include the equilibrium involving the Ti_5_Si_4_ phase, and the isothermal section at 973 K [29] does not contain α_2_-Ti_3_Al. Recently, during an investigation of the dual phase properties of Ti_5_Si_3_ and titanium aluminides, Wu *et al.* [33,34] showed the phase equilibria between Ti_5_Si_3_ phase and γ-TiAl, α_2_-Ti_3_Al phase at 1,473K. Although a limited isothermal phase diagram is available, it has been recognized that the Ti_5_Si_3_ phase is in equilibrium with several titanium aluminides. Therefore, it is useful to calculate the isothermal phase diagram with complete binary thermodynamic data at above 1,273 K. 

In constructing the isothermal phase diagram, the isothermal section at 1,373K has been estimated based upon binary phase information [35,36,37]. Because the free energy values of ternary phases are not available, only binary phases are considered to determine phase equilibria. In this calculation, the standard states were chosen as bcc-Ti, diamond cubic-Si and liquid Al, respectively. The estimated free energy values are shown in Table 1. Since the free energy values of Ti_3_Al, TiAl and TiAl_3_ are described by a superlattice model, the values of the site fraction of atoms in the superlattice indicating minimum free energy values at the given composition were chosen for estimation [37]. Then, the minimum free energy values were determined by adjusting reference reactions. Finally, the various two phase equilibria have been determined by considering the competition of the possible reactions without alloy phase solubility. Based upon this calculation, the isothermal section was determined at 1,373 K as shown in Figure 2. The results of the determination of the isothermal section indicate that the TiSi_2_ phase is not in equilibria with titanium aluminides. However, the Ti_5_Si_3_ phase is in equilibrium with titanium aluminides and thus would provide for a stable reinforcement phase upon formation by an *in situ* reaction, leading that the investigation of the TiSi_2_/TiAl reaction couples is useful for monitoring the formation of Ti_5_Si_3_ in the TiAl phase. 

On the other hand, for the SiC/Me reactions, the properties of the contact materials affect the final diffusion pathway and morphology. The interface reactions of SiC/Metal have been reported and include investigations on the thermal compatibility and/or the solid state reactions [38]. The reaction product sequence and interface morphology in SiC/Metal reactions are mainly dependent on the contact materials. For example, the products of the SiC/Ni reaction show a periodic morphology composed of alternating carbon and silicides [39], but those of the SiC/Cr reaction reveal planar layers of carbides and silicides [21]. While the characterization of individual SiC/Metal interface reactions provides useful information, this is not sufficient to interpret the mechanism governing the SiC/Metal reactions and reaction modes. Some reviews about SiC/Metal reactions regarding to the phase equilibria and phase evolution have been published [40,41,42]. However, it is still necessary to define governing factors and reaction pathways. In fact, the compiled data basis for the SiC/Metal interface reactions can offer a guideline to control the interface properties and phase evolution as well. For example, when the reaction products obtained during interdiffusion processing are not favorable in terms of material properties, a modification of the diffusion pathway can be designed by employing a constructed database to identify a suitable phase combination selection. In the case of the formation of free carbon during SiC/X(I) reaction such as the SiC/Ni combination, the reaction pathway should involve the formation of silicides in order to satisfy the mass balance. 

If the contact material is a strong carbide former such as Mo (affinity of C and metal is high) [43], free carbon is not obtained as a product but rather carbides and silicides. It should be noted that in the diffusion zone the neighboring phase of a carbide or ternary phase is always a silicide. Therefore, it appears that two governing factors—the phase equilibria and mass balance requirements—affect the formation of the product sequence. It has been previously documented that the reaction mode representing the SiC/Me systems has been reported as three different types [42]:

Me + SiC = Silicide + C (graphite)(type I)

Me + SiC = Silicide + Carbide(type II)

Me + SiC = Si + Carbide(type III)

However, with closer examination, since the formation of products in the SiC/Me reaction is governed by a local equilibrium, the formation of Si in the above reactions Type III is not probable based upon the isothermal phase diagram. The recent experimental results about the SiC/Mo and SiC/V reactions which were claimed as type III can be re-examined by the virtue of the current consideration. The diffusion pathways of the SiC/Mo and SiC/V reactions have been reported as follows:

SiC/Mo → SiC/Mo_5_So_3_C/Mo_5_Si_3_/Mo_3_C/Mo [44]

SiC/V → SiC/VC/V_5_Si_3_/V_2_C/V [45]

Based upon the above considerations, it is clear that a type II reaction is equivalent to the type III reaction in terms of the formation of carbides and silicides. The resultant reaction modes representing the SiC/Me systems have been formulated as follows:

Me + SiC → Silicides + C (graphite)(type I)

Me + SiC → Silicides + Carbides + (Me_x_Si_y_C_z_) (type II)

The silicides with free carbon mixtures are products of SiC/X(I) reactions, and the silicides with carbide layers are the products of SiC/X(II) reactions. A ternary phase (Me_x_Si_y_C_z_) is included in parenthesis to generalize the reaction path to include ternary systems with no reported ternary phase such as the Si-C-V system. 

Based upon published isothermal phase diagrams of ternary Si-C-X (X : Ni [46], Ti [47], Mo [48], *etc.*) systems, two general points can be made concerning these systems. First, the solubility of silicides and carbides is limited. In the case of the Si-C-Ni ternary system (850 °C), a limited solubility of silicides and carbide (SiC) has been reported [49]. Second, if a ternary phase exists, it is in equilibrium with one of the carbides and silicides. For example, in the Si-C-Mo system (1,200 °C), the ternary phase (Mo_5_Si_3_C) is in equilibrium with the Mo_2_C and Mo_5_Si_3_ phases [19]. From this comparison, the Si-C-X ternary systems can be separated into type I and type II based upon the shape of the isothermal phase diagram. The main features of type I and type II are the following:

Type I: (1) There is no compound in the Me-C binary system.
  (2) A three phase region (SiC-C-Silicide) exists in the ternary isothermal phase diagram.
Type II: (1) At least one compound exists in the Me-C binary system.
  (2) If a ternary phase exists, it is in equilibrium with a silicide and a carbide (no ternary phase is reported in the Si-C-W [50] and Si-C-V [51] systems).

**Figure 3:**
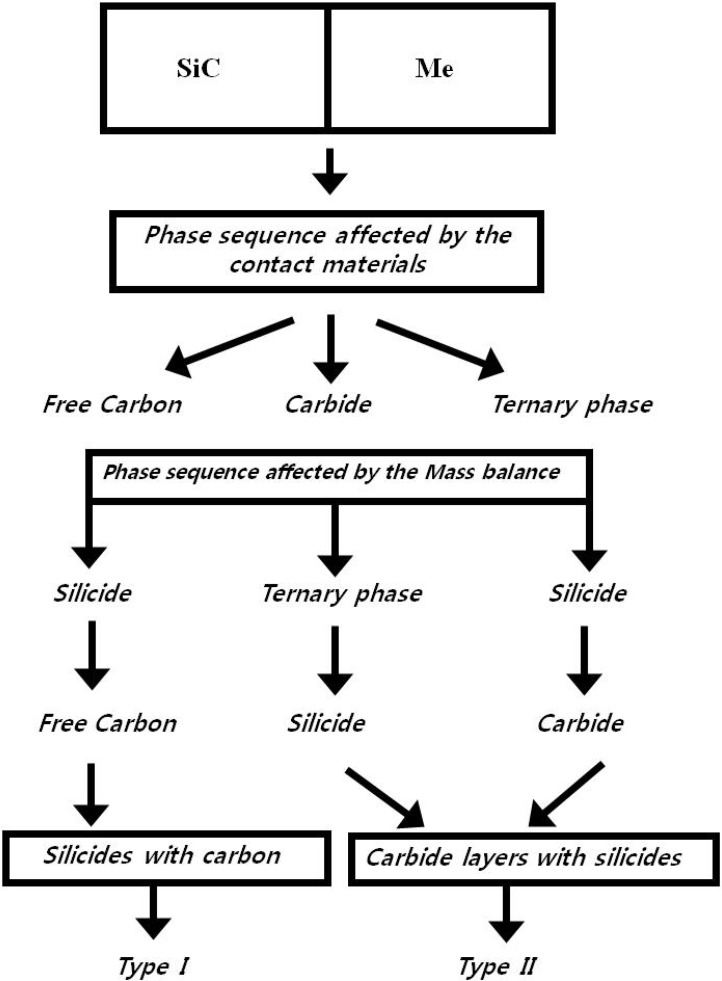
Schematic figure showing the sequence of the products with respect to the contact materials [7].

**Figure 4:**
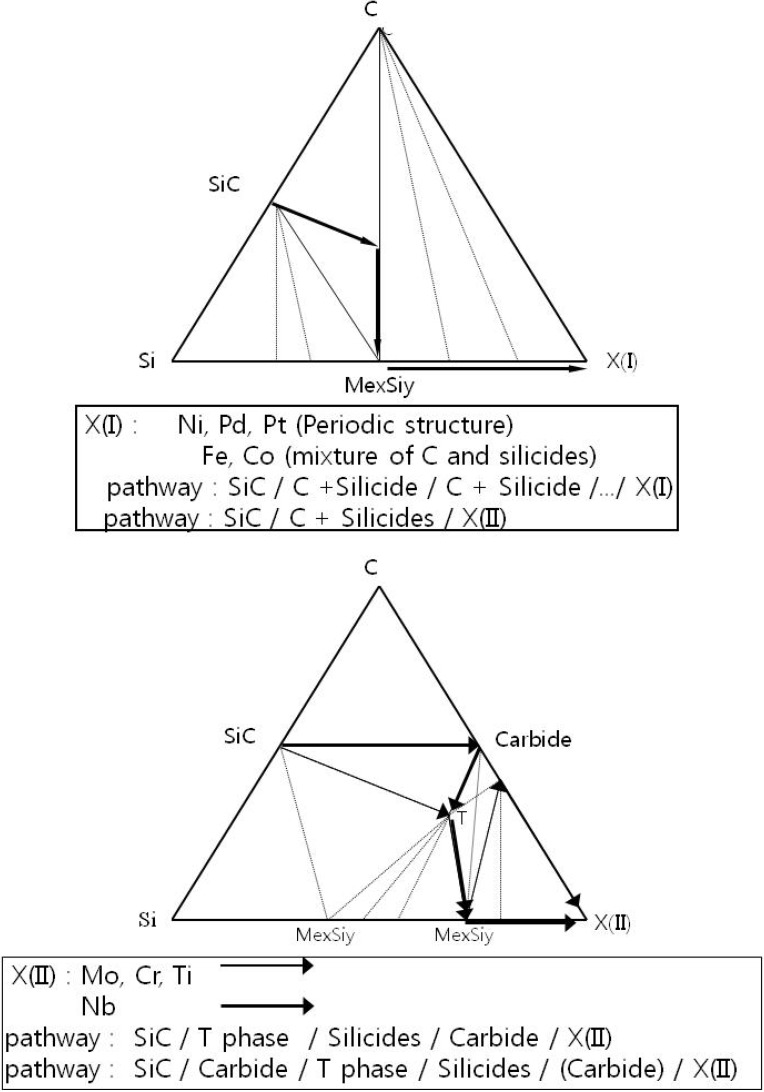
The schematic isothermal phase diagrams and reaction pathway of SiC/X(I) and X(II) [6].

In order to simplify the description of the Si-C-X systems, two schematic ternary isothermal phase diagrams of Si-C-X systems with the diffusion pathway and reaction modes are illustrated in Figure 3 and Figure 4, respectively. The temperature of these diagrams is assumed to be under the melting temperature of any binary or ternary phase. Since the reaction pathway is affected by the phase equilibria, it is meaningful to examine the diffusion pathway based upon this information. 

## 4. Diffusion Pathway in the SiC/Ni and SiC/Cr Systems

SiC/Ni (type I) and SiC/Cr (type II) interface reactions have been examined in detail to identify the entire diffusion pathway. Since both reactions have been well documented in previous works [20,21], efforts have been concentrated on defining the main distinguishing points. It has been reported that the formation of a periodic morphology is due to the difference of individual element mobility. For the SiC/Ni system, Ni is the main moving component, while carbon is immobile [20]. Since the knowledge of the kinetic behavior of components during periodic phase formation is important for understanding the reaction kinetics, SiC/Ni (type I) reaction couples were prepared and investigated. 

### 4.1. SiC/Ni Reactions

**Figure 5:**
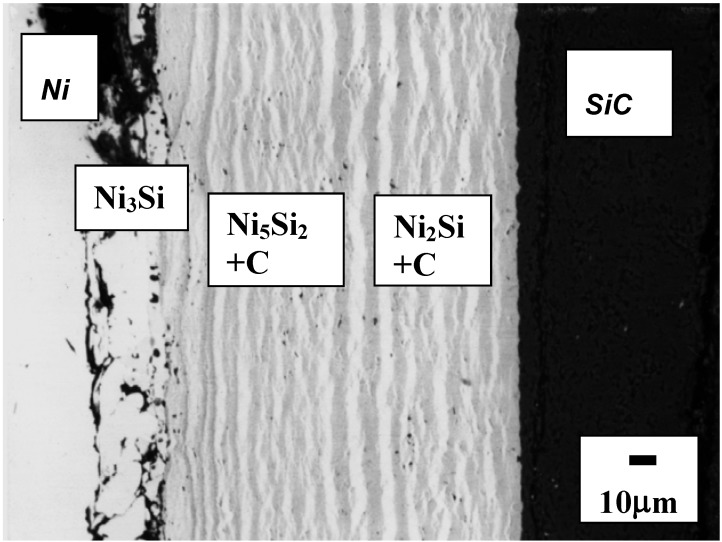
Back Scattered Electron image of Ni/SiC reaction couple annealed at 900 °C for 40 h [6].

The BSE image of a SiC/Ni reaction couple annealed at 900 °C for 40 h is shown in Figure 5. Periodic layers of silicides and carbon were developed as products. The observed product sequence by EPMA is as follows:

SiC/Ni → Ni/Ni_3_Si/Ni_5_Si_2_+C/Ni_5_Si_2_/Ni_5_Si_2_+C../Ni_2_Si+C../SiC.

After diffusion annealing of SiC/Ni reaction couples with markers, van Loo and coworkers [20] reported that the placement of marker has been found at the interface of Ni (one of the end material) and silicides and no traces of carbon or silicon were found on the Ni side. In other words, the Kirkendall marker moved towards the Ni side. If Si or C diffuses over the Ni side, the trace of Si or C should be observed on the Ni side of the Kirkendall marker. Therefore, it can be concluded that the Ni is virtually the only moving component in the SiC/Ni reaction. During the SiC/Ni reaction, Ni diffuses into SiC, and SiC is decomposed into Si and C, and the decomposed Si reacts with Ni to form silicides and free carbon [20]. The presence of the products (silicides and carbon) found at both Ni and SiC side can be understood based upon the vacancy flow. After Ni diffuses toward SiC side, the opposite net flux of the vacancy flow would increase the vacancies of Ni side resulting in the lattice movement toward the Ni side. At the same time, the volume changes will be involved during reactive diffusion. Assuming that Ni and SiC would form the silicides and carbon, the reaction can be made as follows:

10Ni+4SiC → Ni_3_Si+Ni_5_Si_2_+Ni_2_Si+C

Considering the molar volumes of the phases (see Table 1), the volume change for the above reaction is estimated as a 54.9% reduction based upon the initial volume. The large amount of the volume change for the reaction indicates that a stress can develop and should be considered for the SiC/Ni interface reaction. For the development of the periodic layers, two models have been proposed [28]. First, the band, which is formed at the substrate, reaches a critical thickness. Then, the band is lifted from the substrate due to the mechanical stresses accompanying the growth of the band. Then, a new surface (without bands) starts to react and the reaction proceeds during the interdiffusion. The estimated large amount of volume change supports this model. The second model involves the large difference of the component diffusivities. When the first band is formed, one component diffuses fast through the produced band during the interdiffusion. This would produce the product phase (silicide) behind the produced band (carbon + silicides) so that a periodic band morphology develops at the interface. As aforementioned, the Kirkendall markers have been observed at the interface between pure Ni and silicides implying that Ni is virtually the only moving component. This experimental result supports the second model. Therefore, it is considered that both two reaction models operate concurrently to produce the periodic layer morphology. 

**Table 1:** The crystallographic data and molar volumes of Si-C-Ni system [6].

Phase	Pear. Sym.	Prototype	Z	Mol. Vol.
C	hP4	Graphite	4	5.31
β-SiC	cF8	ZnC	8	6.23
Ni	cF4	Al,Cu	4	6.58
β-Ni_3_Si	cP4	L1_2_	4	6.48
Ni_5_Si_2_	hP43	Ni_31_Si_12_	43	6.63
δ-Ni_2_Si	oP12	Co_2_Si	12	6.59
θ-Ni_2_Si	hP6	Ni_2_In	6	6.33
NiSi	oP8	MnP	8	7.32
NiSi_2_	cF12	CaF_2_	12	7.93
Si	cF4	C(Dia.)	8	12

### 4.2. SiC/Cr Reactions

The product phase sequence of SiC/Cr in the temperature range between 1,000 °C and 1,200 °C has been reported as follows [17]:

SiC/Cr → SiC/Cr_5_Si_3_C/Cr_7_C_3_+Cr_3_Si/Cr_7_C_3_/Cr_23_C_6_/Cr

It should be noted that the carbide layers are produced next to the Cr side, indicating that the diffusion distance of carbon from the SiC is larger than that of Si in SiC. 

## 5. Diffusion Pathway Analysis and Chemical Potential Diagram

The various composite design features emphasize the importance of the diffusion path that is followed during reaction in multicomponent systems. The prediction of the diffusion path from theory requires detailed knowledge of the diffusivities for each component, including cross terms, as well as a complete phase diagram including two phase field tie lines. For a ternary system this means that information on diffusivities is available for each phase. Even though there are constraints on the diffusivities that can be applied to reduce the data base requirements, the necessary information for theoretical prediction is still large and generally unavailable for given multicomponent systems. In order to provide some theoretical guidance to judge the diffusion path trajectory, alternative approaches have been proposed based upon simplified conditions [52,53]. All analysis methods include two basic requirements of diffusion path. First, the pathway should cross the line connecting the end member compositions in order to satisfy mass balance. Secondly, a stable path should follow the isothermal phase diagram, and coincide with two-phase field tie lines to satisfy local equilibrium. Accordingly, the accumulated knowledge on the diffusion pathway can provide a strategy for materials selection during phase evolution. 

**Figure 6:**
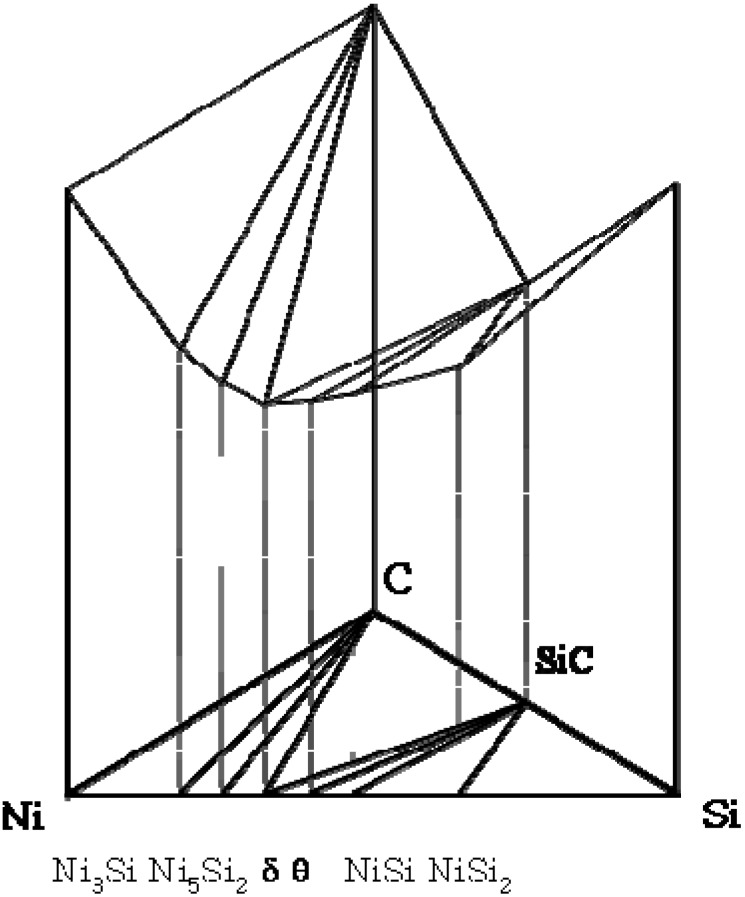
Schematic 3-dimensinal chemical potential diagram of the Si-Ni-C ternary system at 900 °C [6].

**Figure 7:**
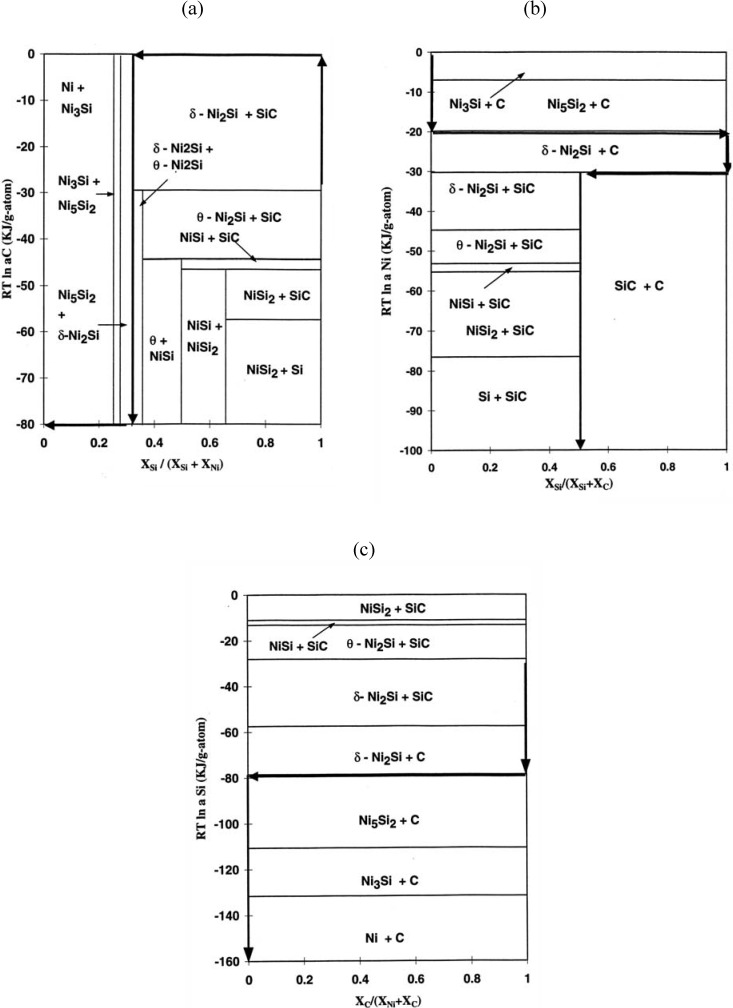
The chemical potential diagrams with diffusion pathway of the Si-C-Ni system (a) with respect to carbon, (b) with respect to nickel, (c) with respect to silicon [6].

Since the formation of product phases is affected by the chemical potential, it is useful to consider the observed diffusion pathway within the chemical potential framework [26]. While the chemical potential diagrams of the SiC/Cr reaction are accessible [21], it is useful to examine the SiC/Ni reaction. Chemical potential diagrams with respect to Si, C and Ni have been constructed based upon the free energy values of each phase, assuming that each phase is a line compound (*i.e.*, the solubility of each phase is negligible). From the estimated values of binary systems the chemical potential values in the ternary system has been estimated with a graphical method. The resulting activity values of ternary regions are shown in Table 2. The schematic three dimensional diagram based upon these values is illustrated in Figure 6. Each plane is the tangential plane at the associated free energy surface. It should be noted that each three phase equilibrium plane of the isothermal phase diagram corresponds to each tangential plane. The extension of the tangential plane to the three axes indicates the chemical potential values. For example, the extension of the Ni_3_Si +Ni_5_Si_2_ +C plane to the axes indicates the chemical potential values of Ni, Si and C (Figure 7). Based upon this three-dimentional diagram, the chemical potential diagrams with respect to Ni (Figure 7a), Si (Figure 7b) and C (Figure 7c) have been constructed at 900 °C. Also, the diffusion pathway of SiC/Ni is marked in the stability diagrams. While the chemical potentials of Si and Ni decrease, that of carbon increases and decreases through the reaction layers by forming free carbon. For the SiC/Cr reaction, it has been reported that the chemical potentials of Si, C and Cr decrease through the reaction layer (see [20] for component chemical potential diagrams). Since for type I reactions, the chemical potential of carbon increases and decreases through the reaction zone due to the production of free carbon from SiC, the identification of the chemical potential variation is also a characteristic feature. Therefore, the chemical potential behavior appears to be related to the kinetic difference of reaction modes between the Ni (type I) and the Cr (type II). 

**Table 2:** The estimated activity values of each component in a designated ternary equilibrium plane at 900 °C [6].

Equilibrium Planes	Activity values of components		
	Ni	Si	C
**Ni+Ni_3_Si+C**	1.00	7.56E-07	1.00
**Ni_3_Si+Ni_5_Si_2_+C**	4.72E-01	7.17E-06	1.00
**Ni_5_Si_2_+δ-Ni_2_Si+C**	1.20E-01	2.22E-04	1.00
**δ-Ni_2_Si+SiC+C**	3.92E-02	2.14E-03	1.00
**δ-Ni_2_Si+θ-Ni_2_Si+SiC**	8.33E-03	4.97E-02	4.31E-02
**θ-Ni_2_Si+NiSi+SiC**	3.39E-03	2.46E-01	8.70E-03
**NiSi+NiSi_2_+SiC**	2.68E-03	3.11E-01	6.88E-03
**NiSi_2_+SiC+Si**	2.77E-04	1.00	2.14E-03

**Figure 8:**
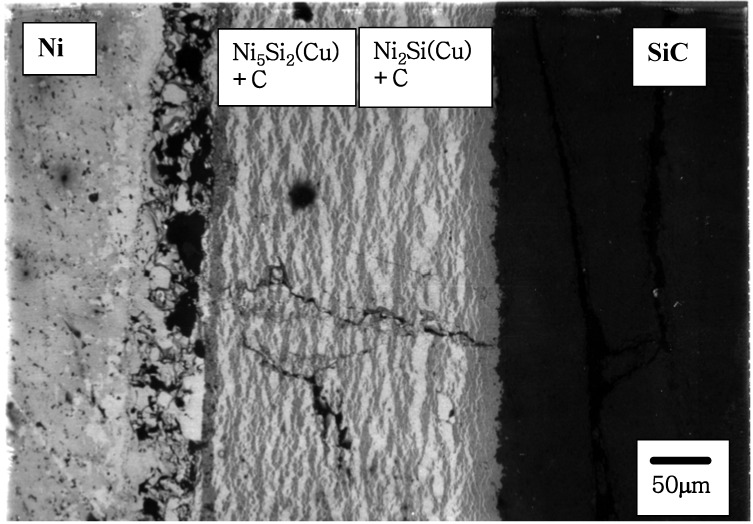
BSE image of Ni/Cu/SiC reaction couple annealed at 900 °C for 40 h [6].

When the reaction extent can be controlled, a thin interface debonding layer can have a beneficial effect on the overall fracture toughness of a composite [54]. The debonding mechanism has been reported that a produced carbon layer can provide a crack deflection site due to the weak strength of carbon at the interface [54]. However, it has also been reported that the reaction kinetics of SiC/Ni is fast during isothermal annealing at (and above) 1,000 °C, resulting in a severe degradation of SiC [55]. This indicates that the control of the periodic layer is placed in the central issue for controlling overall materials properties. In order to examine the effect of a biasing layer (excess flux), a selected biasing interlayer was inserted into a SiC/Ni diffusion couple subsequently annealed at 900 °C for 40 h. Cu (X(I)) and Cr (X(II)) have been selected as interlayers based upon the proposed reaction classification. Since the SiC/Ni reaction represents a type I reaction, the application of a type II component such as Cr would change the carbon flux by producing carbides based upon the above discussion. Indeed, when a Cu interlayer (25 μm) is inserted in a SiC/Ni reaction couple, the products are obtained with a periodic layer morphology (Figure 8). 

**Figure 9:**
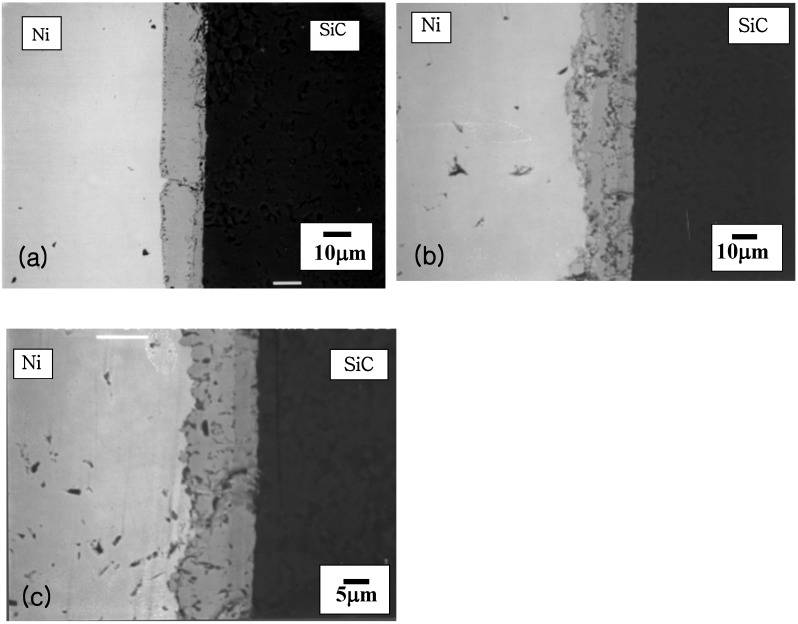
BSE image of Ni/Cr/SiC reaction couple annealed at 900 °C for (a) 40 h, (b) 80 h and (c)120 h [6].

The reaction product sequence was observed as: Ni/Ni_2_Si(Cu)+C/Ni_5_Si_2_(Cu)+C/Ni. It should be noted that the diffusion of Cu (interlayer) into both sides is fast, so that the diffusion pathway is moved into the Ni rich side. However, when a Cr interlayer (Cr was sputter-deposited with a nominal thickness of 10 μm) is placed in a SiC/Ni reaction couple, the morphology of the reaction product layers is planar [Figure 9 (a)]. The product sequence identified by EPMA is Ni(s.s)/Cr/Cr_23_C_6_/Cr_7_C_3_/T(Cr_5_Si_3_C)/SiC. The diffusion of Cr is relatively stable compared to the Cu layer and the reaction products are distinctly biased by the excess flux of Cr. In order to investigate the phase evolution of Ni/Cr/SiC reaction couple with respect to the longer anneal time (>40 h), the same reaction couples were annealed 80 h and 120 h at the same temperature. The BSE image (the Ni/Cr/SiC reaction couple annealed at 80 h at the same temperature) is shown in Figure 9(b). The product sequence is observed as Ni(s.s)/Cr_23_C_6_/Cr_7_C_3_/T((Cr,Ni)_5_Si_3_C/SiC. It should be noted that the inserted Cr layer disappeared and Cr carbide layers are produced, indicating the dominant diffusion of carbon to the Cr layer. Also, Ni diffuses towards the SiC side, resulting that the solubility of Ni on the ternary phase (Cr_5_Si_3_C) becomes up to about 10 at%. Also, the BSE image of Ni/Cr/SiC reaction couple annealed at 120 h at the same temperature is shown in Figure 9 (c). The diffusion pathway is obtained as Ni(s.s)/Cr_7_C_3_/T((Cr,Ni)_5_Si_3_C/SiC. The solubility of Ni on the ternary phase (Cr_5_Si_3_C) is observed up to 13 at %. Also, the concentration of Ni increased and decreased through the (Cr,Ni)_5_Si_3_C phase, indicating that the Ni diffusion was fast enough to reach the SiC phase. However, the low solubility of Ni on SiC may disturb the Ni diffusion towards the SiC side. It should be noted that the Cr_23_C_6_ phase is not observed in this reaction couple. This may be due to the insufficient amount of Cr to maintain the Cr_23_C_6_ phase during interdiffusion reaction, so that the diffusion pathway is moved away from Cr. It is clear that the diffusion pathway moves away from the Cr side by increasing the annealing time. For the Cr biased reaction couples, the reaction layers were clearly observed as a planar shape (instead of periodic layers) within experimental kinetic bounds providing a clear evidence for controlling interface morphology strategies. 

## 6. Interface Reactions of TiAl/TiSi_2_

**Figure 10:**
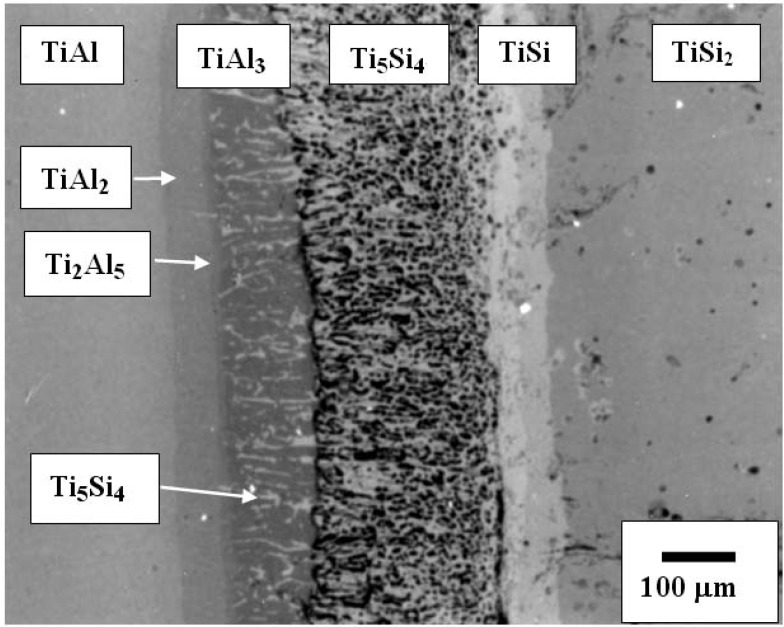
Back Scattered Electron (BSE) image showing TiAl/TiSi_2_ reaction couple annealed at 1,373 K for 200 h [7].

As aforementioned, another excellent example for useful practice of diffusion pathway anaylsis is the Ti-Al-Si ternary system. The back scattered image of TiSi_2_*/*γ-TiAl annealed at 1,100 °C for 200 h is shown in Figure 10. Several phases including a porous layer developed at the interface of TiSi_2_ and TiAl. The phase sequence was obtained as TiAl/TiAl_2_/Ti_2_Al_5_/TiAl_3_ + Ti_5_Si_4_/Ti_5_Si_4_/TiSi/TiSi_2_. An irregular geometry of the Ti_5_Si_4_ phase was developed over a 100 μm layer thickness, in which the dark regions were observed as pores. It is noted that the Al solubility in the silicides (*i.e.*, Ti_5_Si_4_ and TiSi) was detected to be less than 2 at %. However, the solubility of Si in the TiAl_3_ phase was detected to range up to 7 a t%, which is in agreement with previous results in which the large solubility of silicon in the TiAl_3_ phase has been reported [23]. 

**Figure 11:**
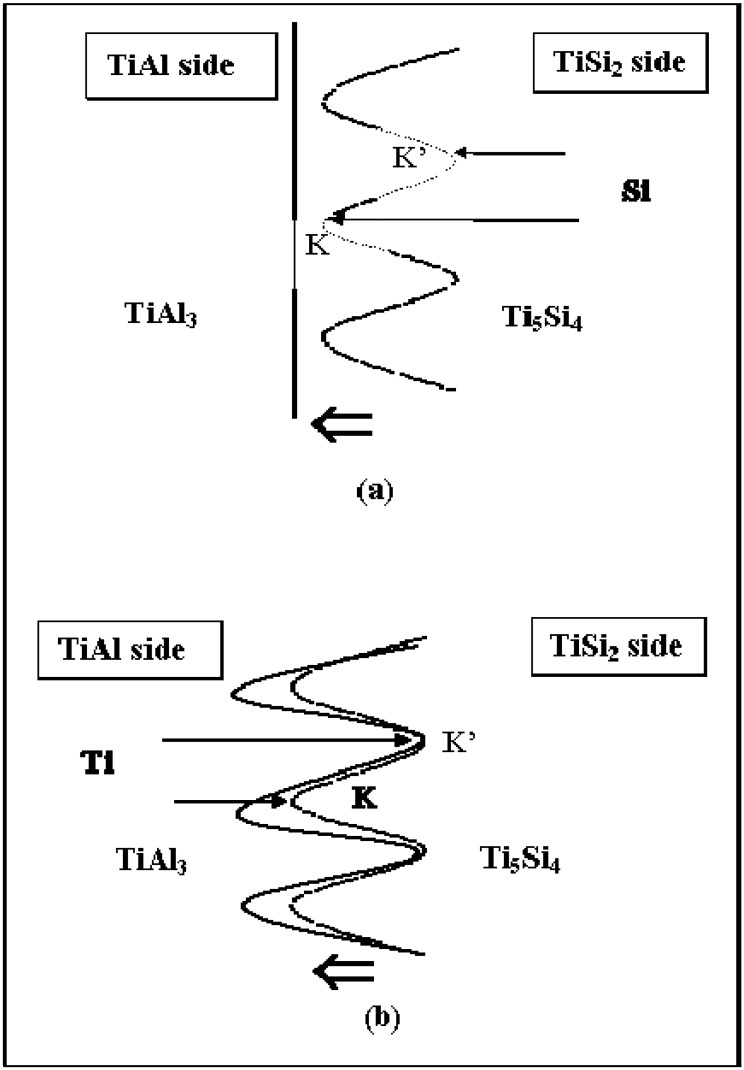
The development of the planar or columnar interface with respect to the rate limiting component (a) the decay of the pertubation when the rate limiting component is Si (The feeding rate to the point **K’** is faster than that to the point **K** (b) the development of pertubation when the rate limiting component is Ti (The arriving rate to the point **K** is faster than that to the point **K’**) [7].

The information about partial kinetics can be obtained qualitatively from the formation of the columnar Ti_5_Si_4_ phase developed in the TiAl_3_ phase. In order to evaluate the kinetics of the movement of components from the resultant morphology, the rate limiting component has been evaluated. When initial driving force for an interdiffusion reaction is a composition difference, the Ti and Al atoms would move towards the TiSi_2_ side, but Si would diffuse in the direction of the TiAl side. The columnar zone of the Ti_5_Si_4_ phase grows towards the TiAl side as shown in **Figure 11**. If Si of the Ti_5_Si_4_ phase is the rate limiting component (D _Ti(TiAl3)_ > D _Si(Ti5Si4)_), the feeding of Si atoms to the columnar Ti_5_Si_4_ phase determines the shape of the interface. Since the interface moves towards the TiAl side, the feeding rate to the point **K’** is faster than that to the point **K**. Then, the pertubation would decay due to the faster movement of point, resulting that the planar interface develops. However, if Ti of the TiAl_3_ phase is the rate limiting component (D _Ti(TiAl3)_ < D _Si(Ti5Si4)_), the arriving rate to the columnar Ti_5_Si_4_ phase establishes the shape of the interface. In this case, the arriving rate to the point **K** (the tip of the columnar Ti_5_Si_4_ phase) is faster than that to the point **K’**, resulting in the development of pertubations. Judging from the columnar morphology of the Ti_5_Si_4_ phase in Figure 10, the columnar Ti_5_Si_4_ phase grows through the TiAl_3_ phase, indicating that the rate limiting component is Titanium (D _Ti(TiAl3)_< D _Si(Ti5Si4)_). 

**Figure 12:**
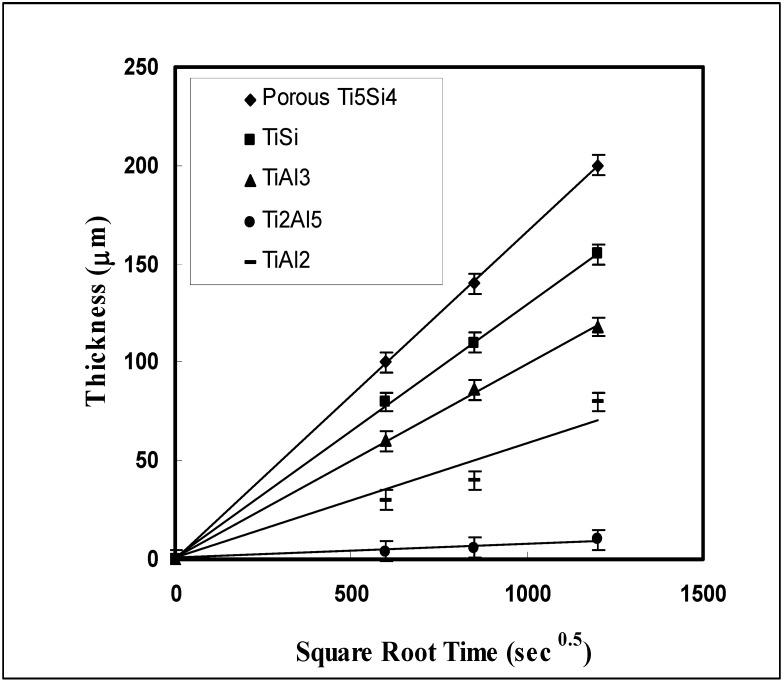
The growth of each product phase of TiSi_2_/TiAl reaction couples annealed at 1,373 K [7].

To obtain the kinetic mode of each phase, the thickness (ξ) of the product phases was examined as a function of the annealing time. When the intermediate phases grow by diffusion control, the kinetics follow a parabolic law (ξ^2^ = k t). The estimated results show that the thickness (ξ) of the intermediate phases followed the parabolic law (Figure 12). Compared with the growth rate of other phases, the growth rate of Ti_5_Al_4_ showed the largest sensitivity to the annealing time. Even though the Ti_5_Si_4_ phase was developed as a porous structure, the growth rate shows the same order of magnitude as that of the TiSi and TiAl_3_ phases, and the growth rate of the Ti_2_Al_5_ phase showed the lowest among the product phases. 

Since the titanium aluminide products show a limited Si solubility, it is useful to compare them with the growth in the binary system. It has been reported that most of the intermetallic phases such as Ti_3_Al, TiAl, TiAl_2_ and Ti_2_Al_5_ do not follow a parabolic law due to the influence of grain boundary diffusion with an exception of the TiAl_3_ phase which follows a parabolic law [57,58]. However, Hirano and Iijima [59] reported that as a result of diffusion annealing of β-Ti*/*TiAl couples, the Ti rich product phases (α-Ti, β-Ti and Ti_3_Al) are controlled by volume diffusion (following the parabolic law) at long annealing time (more than 200 h). Also, it has been pointed out that the reason for grain boundary diffusion in the Ti-Al system is due to the short annealing time [59]. Therefore, based upon the above results, it is considered that when the reaction couples are examined at long term annealing, the parabolic growth mode is also dominant in the binary Ti-Al system. In this study, all of the product phases followed a parabolic law in agreement with the previous report [59]. 

The growth kinetics of the silicide formation also has been examined by Cockeram *et al.* [60]. The parabolic rate constants of TiSi and Ti_5_Si_4_ phase have been estimated as 1.19 × 10^-10^ cm^2^/sec and 5.44 × 10^-11^ cm^2^/sec, respectively, after Ti/Si diffusion annealing at 1,373 K. While it has been reported that the growth rate of TiSi was larger than that of Ti_5_Si_4_ [58], the current study shows that the growth rate of Ti_5_Si_4_ was larger than that of the TiSi phase. In fact, the estimated rate constants of the TiSi and Ti_5_Si_4_ phase were obtained as 1.58 × 10^-10^ cm^2^/sec and 2.67 × 10^-10^ cm^2^/sec respectively, indicating that the growth rate of Ti_5_Si_4_ has been increased about one order of magnitude higher than that of the previous report [60]. It seems that this behavior is due to the effect of the third element (the effect of Al on the growth of Ti_5_Si_4_ phase). 

In the reactive diffusion, the thicknesses of the product phases are related to the interdiffusion coefficients. However, when an intermetallic compound with a very narrow homogeneity range forms during reactive diffusion, the measurement of a concentration gradient is virtually not possible, because the concentration gradients of components does not develop clearly. In order to evaluate the diffusivity for an intermetallic compound having a limited solubility, Wagner [61] introduced an integrated diffusion coefficient, D_int_, which is defined as an integrated value of the interdiffusion coefficient over its (unknown) limits of homogeneity between N’ and N” (mole fraction) and expressed as follows:

(4) $$D_{\text{int}}=\int_{N^{\prime }}^{N^{\prime \prime }}\tilde{D}dN$$

For the case of producing several phases with a very narrow homogeneity region, the integrated diffusion coefficient of phase *i* can be written as in Equation (5):

(5) $$D_{\text{int}}^{i}=A+B$$

where $A=\left(N^{i}-N^{-}\right)\frac{\left(N^{+}-N^{i}\right)}{N^{+}-N^{-}}\frac{{\left(\text{Δ}x^{i}\right)}^{2}}{2t}$ and:

$$B=\frac{\text{Δ}x^{i}}{2t}*\left[\frac{\left(N^{+}-N_{i}\right)\sum_{\upsilon =2}^{\upsilon =i-1}\frac{V_{m}^{i}}{V_{m}^{\upsilon }}\left(N^{\upsilon }-N^{-}\right)\text{Δ}x^{\upsilon }+\left(N^{i}-N^{-}\right)\sum_{\upsilon =i-1}^{\upsilon =n-1}\frac{V_{m}^{i}}{V_{m}^{\upsilon }}\left(N^{+}-N^{\upsilon }\right)\text{Δ}x^{\upsilon }}{N^{+}-N^{-}}\right]$$

N^+ (-)^: mole fraction at +∞ (-∞)
V_m_: molar volume
Δx^i^: thickness of i phase
t: time

**Figure 13:**
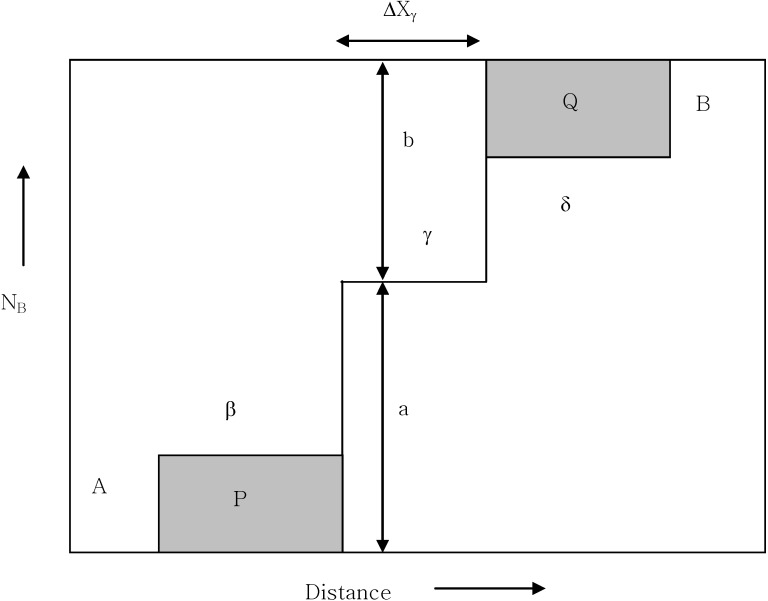
The schematic concentration profile of A/B diffusion couple produced β, δ and γ [7].

Based upon the above equation, the integrated diffusivity of phases can be obtained if thicknesses and compositions are known. Assuming that the molar volume does not change from one phase to another, Equation (5) can be expressed in a simpler form [20]. For the case of an A/B reaction couple producing three line compounds, α, γ and δ, the integrated diffusivity of γ is written as Equation (6):

(6) $$D_{\text{int}}^{\gamma }=\left[\frac{a\times b}{a+b}\right]*\frac{\text{Δ}x_{\gamma }^{2}}{2t}+\frac{\text{Δ}x_{\gamma }}{2t}*\left[\frac{b\times P+a\times Q}{a+b}\right]$$

where P and Q equal the hatched areas in Figure 13 and a = N_A_^γ^ - N_A_^-^ and b = N_A_^+^ - N_A_^γ^, respectively. 

The integrated diffusivity of the current reaction couple has been estimated based upon the observed product layer thickness. When applying the above equation, the porous layer has been approximated as a solid layer as a line compound. The estimated parameters of Equation 3 and the integrated diffusion coefficient are shown in Table 3. The diffusion coefficient of Ti_2_Al_5_ marks the lowest value. Since the integrated diffusion coefficient corresponds to the integral values of the interdiffusion coefficient as defined previously, it is considered that the low value of Ti_2_Al_5_ is due to the relatively small thickness of the phase (ΔX). 

**Table 3:** The estimated integrated diffusion coefficients and the related parameters.

Phases	Parameter ‘a’	Parameter ‘b’	Area	Area	D^int^
			P	Q	(m^2^/sec)
TiSi	0.5	0.16	6.30 × 10^-5^	-	1.76 × 10^-15^
Ti_5_Si_4_	0.45	0.21	-	1.60 × 10^-5^	2.78 × 10^-15^
TiAl_3_	0.25	0.25	1.27 × 10^-4^	6.39 × 10^-6^	4.79 × 10^-15^
Ti_2_Al_5_	0.286	0.214	1.48 × 10^-4^	5.10 × 10^-6^	2.58 × 10^-16^
TiAl_2_	0.33	0.17	1.50 × 10^-4^	-	1.31 × 10^-15^

**Figure 14:**
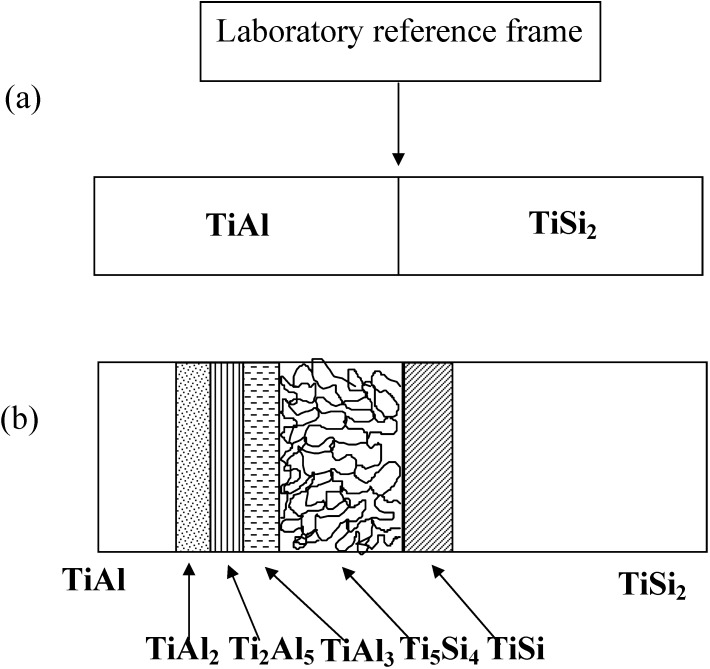
The schematic illustration of the reaction products and the placement of the laboratory reference frame (not put to a scale) (a) before reaction, (b) after reaction.

Since the sequence of the formation of the product phases are closely related to the movement of components, it is useful to examine the kinetic features of the product phases. The schematic figure of the reaction products is given in Figure 14. It should be noted that the TiAl_2_, Ti_2_Al_5_, TiAl_3_ and porous Ti_5_Si_4_ were observed at the TiAl side, but TiSi formed at the TiSi_2_ side based upon the initial placement of the interface (laboratory reference frame). 

Under an assumption that the concentration difference is the initial driving force, Si of the TiSi_2_ phase would diffuse towards the TiAl side and both Ti and Al of the TiAl phase would move towards TiSi_2_ side. The diffusion of Si produces the TiSi phase (lower Si concentration) on the TiSi_2_ side. Also, the movement of Si is the reason for the formation of the Ti_5_Si_4_ phase, since Si is the only source of the silicides for this reaction couple. The formation of the porous Ti_5_Si_4_ phase on the TiAl side implies that the movement of Si in the TiSi_2_ phase is faster than that of Ti in the TiAl. Otherwise, the porous Ti_5_Si_4_ phase would form in the TiSi_2_ side. The higher Ti concentration of the porous Ti_5_Si_4_ phase than that of the TiAl phase indicates that Ti of TiAl diffuses towards the Ti_5_Si_4_ phase on the TiAl side, resulting in the formation of Al rich products next to the porous Ti_5_Si_4_ phase such as TiAl_2_, Ti_2_Al_5_ and TiAl_3_ on the TiAl side. Regarding to the diffusion behavior of Al, it should be noted that the porous Ti_5_Si_4_ phase which has little solubility of Al is produced in the TiAl side. Since Al of TiAl existed initially (later occupied by the Ti_5_Si_4_ phase) and the reaction front of the porous Ti_5_Si_4_ phase moves towards the TiAl side, there are two possibilities to develop the porous Ti_5_Si_4_ phase. (a) When the Ti_5_Si_4_ phase grows towards the TiAl side, Al is trapped by the Ti_5_Si_4_ phase, finally evaporated and the traces of Al are observed as pores. (b) Since Al can not diffuse towards the Ti_5_Si_4_ phase (low solubility of Al in the Ti_5_Si_4_ phase), the excess Al does diffuse backwards. Finally, Kirkendall voids due to this flux imbalance initiate the development of pores. 

**Figure 15:**
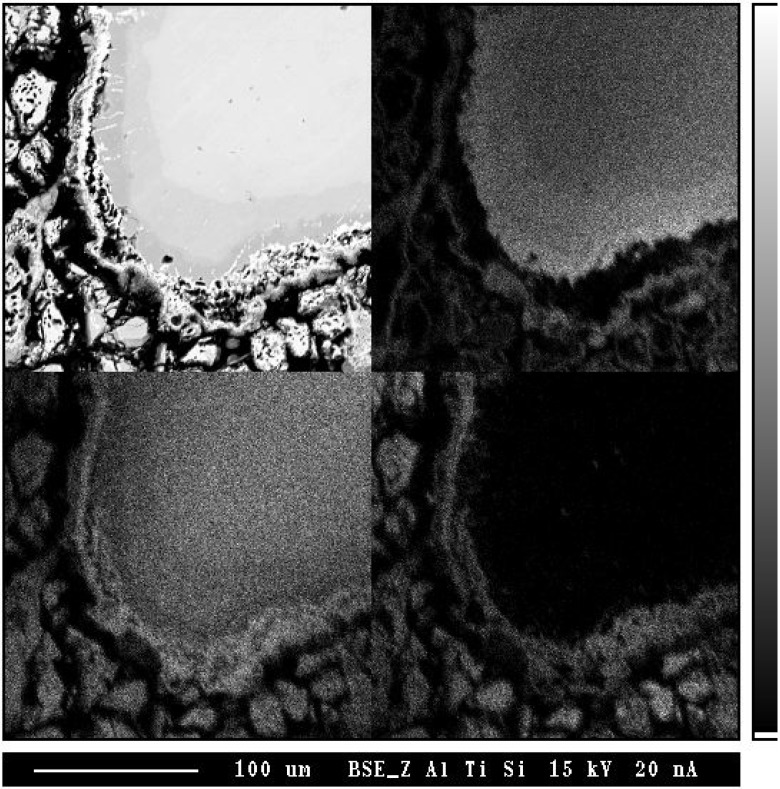
The BSE image (a) and X-ray maps (with respect to (b) Al, (c) Ti and (d) Si) of TiAl/TiSi_2_ powder mixture annealed at 1,100 °C for 40 h [7].

In order to investigate the formation mechanism of the porous layer, cold pressed powder mixtures of TiSi_2_ and TiAl were vacuum sealed and annealed at 1,100 °C for 30 h followed by an air cooling (Figure 15). Several products and porous Ti_5_Si_4_ phase were observed, which is consistent with the TiSi_2_/TiAl reaction couple. While porous layers were found, a clear evidence for the traces of an excess Al has not been found. Therefore, it can be drawn that the second mechanism (backward diffusion of Al) is a main reason for the formation of the porous phase, which is in agreement with the previous results [62]. Also, for the reactive diffusion reaction, volume changes are usually involves during interdiffusion, which might contribute to form pores at the interface of TiAl_3_ and Ti5Si_4_ phase together with flux imbalance. 

**Figure 16:**
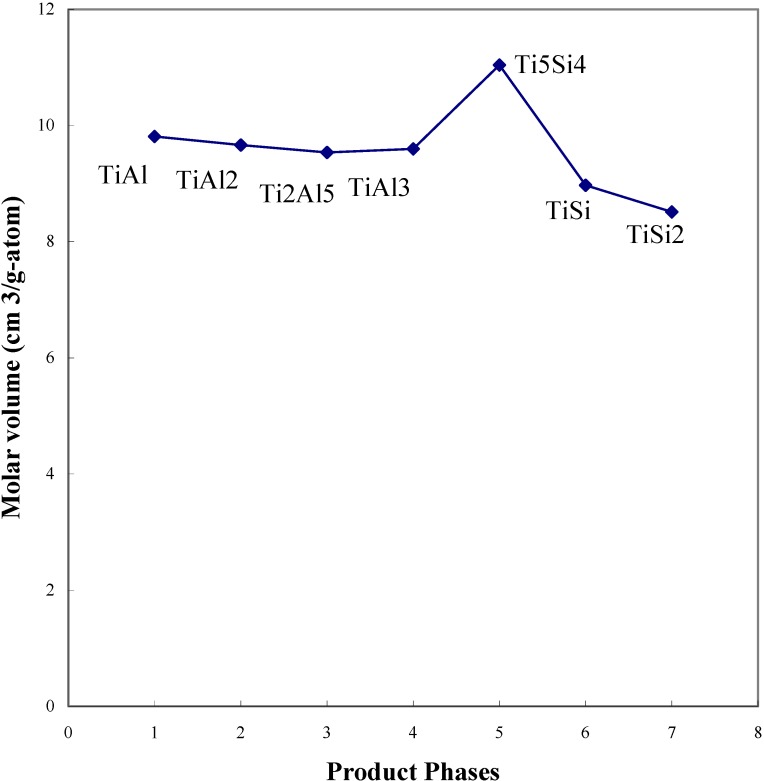
The molar volumes of the product phases of the TiAl/TiSi_2_ reaction couple [7].

In diffusion controlled reactions, each product layer grows with respect to the relative flux of neighboring phases accompanied by volume changes, resulting in the development of stress between the product phases. In order to estimate the volume change during interdiffusion processing, the binary lattice constants were employed to calculate the molar volumes, considering the low solubility of the third element in the binary compounds. The structures, volumes of the unit cells and the molar volumes of the phases in the Ti - Si and Ti-Al system are shown in Table 4. Compared to the molar volumes of the product phases, the molar volumes of the Ti_5_Si_4_ and Ti_5_Si_3_ phases showed relatively high values. Also, the molar volume of each phase is shown based upon the observed diffusion path of TiSi_2_/TiAl (Figure 16). It is clear that the molar volume of the Ti_5_Si_4_ phase is relatively high compared to the TiAl_3_ and TiSi phases. 

**Table 4:** The Crystal structures and molar volumes of the Titanium alluminides and Titanium silicides (V: volume of the unit cell, Z : Number of formula units in the unit cell) [7].

Phase	Pear.sym.	prototype	V(nm^3^)	Z	Mol. Vol.
					(cm^3^/g-atom)
β-Ti	cI2	W	0.0317	2	9.543
TiAl	tP4	AuCu	0.0652	4	9.813
TiAl_2_	tI24	Ga_2_Hf	0.3850	24	9.667
Ti_2_Al_5_	tI16	Al_3_Zr	0.2540	16	9.539
TiAl_3_	tI8	Al_3_Ti	0.1276	8	9.598
(Si)	cF8	C(dia)	0.1600	12	12.000
TiSi_2_	oF24	C54	0.3394	24	8.513
TiSi	oP8	TiSi	0.1192	8	8.970
Ti_5_Si_4_	TP36	Si_4_Zr_5_	0.6602	36	11.040
Ti_5_Si_3_	hP16	Mn_5_Si_3_	0.2835	16	10.670
Ti_3_Si	tP32	Ti3P	0.5280	32	9.933
(Al)	cF4	Cu	0.0664	4	9.667

The volume changes and free energy changes of the relevant reactions are shown in Table 5. The volume change ratios were calculated from the ratio of total product molar volumes to the total initial volumes. It should be noted that most of the reactions showed a large volume reduction. Especially, reaction (2) showed the largest value of the volume reduction ratio by producing Ti_5_Si_4_ phase from TiSi and Ti. However, reaction (6) showed a large volume expansion by producing Ti_5_Si_4_ from TiAl_3_ and Si. From the calculated results, it can be estimated that a large volume change is involved at the interfaces of the Ti_5_Si_4_ phase. It is considered that since Ti_5_Si_3_ is placed in the Ti rich side of the Ti-Si binary system, the flux of Ti is not sufficient to allow for the formation of Ti_5_Si_3_. In order to produce the Ti_5_Si_3_ phase, the interdiffusion reaction with the additional Ti flux has been investigated. 

**Table 5:** The calculated volume change ratio and reactions.

No	Reactions	Volume changeRatio	Free energy of Reaction
		(%)	(KJ/mol)
1	TiSi_2_ + Ti = 2TiSi	-6.6	-130.7
2	4TiSi + Ti = Ti_5_Si_4_	-75.7	-61.8
3	TiAl + Al = TiAl_2_	-50.4	-21.4
4	2TiAl_2_ + Al = Ti_2_Al_5_	-67.1	-14.1
5	Ti_2_Al_5_ + 2Al = 2TiAl_3_	-33.5	-12.0
6	5TiAl_3_ + 4Si = Ti_5_Si_4_ + 15Al	+62.6	-269.2

## 7. Control of the Interface Reactions in TiSi_2_/Ti/TiAl

**Figure 17:**
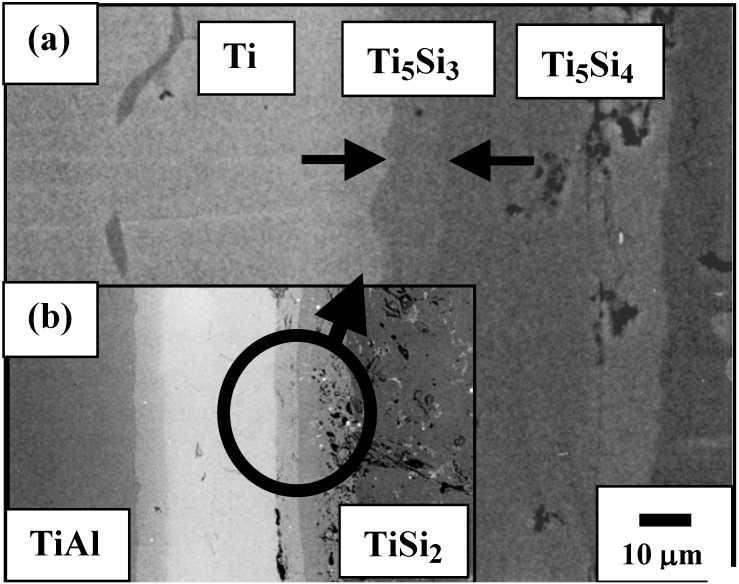
BSE image of TiSi_2_/Ti/TiAl reaction couple annealed for 1,100 °C for 400 h [7].

In order to investigate the stability of the biased reaction, the TiSi_2_/Ti/TiAl couples were annealed at 1,100 °C for 400 h (Figure 17). It should be noted that the porous Ti_5_Si_4_ phase was not developed in the biased diffusion couple, and the expected Ti_5_Si_3_ phase was successfully produced. For the case of the 200 h annealed sample (not shown), the thickness of the Ti_5_Si_3_ and Ti_5_Si_4_ phases were about 10 μm and 40 μm respectively. Compared to the reaction couples annealed for 200 h and for 400 h, it should be noted that the thickness of the β-Ti phase is reduced from about 300 μm (200 h annealed couple) to about 260 μm (400 h annealed couple). The concentration of Al in the Ti_5_Si_3_ phase was detected as about 5 at % and the solubility of the Al in the Ti_5_Si_4_ phase was extremely small, which is the same result as the TiSi_2_/TiAl reaction couple. Moreover, it is clear that the concentration of the Al is increased in the Ti_5_Si_3_ phase, then abruptly decreases in front of the Ti_5_Si_4_ phase. This concentration variation of the Al indicates two possibilities. Since the solubility of Al in the Ti_5_Si_4_ phase is low, the Ti_5_Si_4_ phase serves as a barrier for the diffusion of the Al, resulting in the pile up of the Al in front of the Ti_5_Si_4_ phase. The other possible reason for the concentration variation is due to the nature of the diffusion pathway in a ternary system (*i.e.*, concentration up and down during the interdiffusion in a ternary system). 

In the modified reaction couples, the Ti_5_Si_3_ phase was produced and the pores of the Ti_5_Si_4_ phase disappeared as well. The porous phase appeared at the interface of the TiAl_3_ and Ti_5_Si_4_ phases due to the flux imbalance associated with volume changes. Moreover, the interface shape of the Ti_5_Si_4_ and β-Ti was observed as planar. Therefore, it is concluded that the pores and the morphology can be controlled by applying the modified reaction. 

## 8. Reaction Pathway Analysis of the Ti-Al-Si System

The direction of reaction pathway can be analyzed qualitatively by considering both mass balance and reaction barrier effects. In the reactive diffusion of the TiSi_2_ and TiAl, only Si atoms in the TiSi_2_ phase would move towards the TiAl phase side, however, both Ti and Al atoms in the TiAl phase would diffuse towards the TiSi_2_ phase side due to the initial concentration difference. For the Si diffusion in the TiSi_2_ phase, only Si atoms would move towards the TiAl phase, resulting in the formation of the titanium rich phases such as the Ti_5_Si_3_, Ti_5_Si_4_ and TiSi in the TiSi_2_ side. Therefore, in order to satisfy mass balance, aluminum rich phases such as TiAl_3_ and Ti_2_Al_5_ should form on the TiAl phase side of the reaction couple. As a result of the diffusion reaction of TiSi_2_ and TiAl, the actual product phases were obtained as follows:

TiAl / TiAl_2_ / Ti_2_Al_5_ / TiAl_3_ / Ti_5_Si_4_ / TiSi / TiSi_2_ (Al rich)   (Si rich)

From the produce phase developed, the Si rich product phases determine the initial direction and the Al rich product phases satisfy the mass balance. 

**Figure 18:**
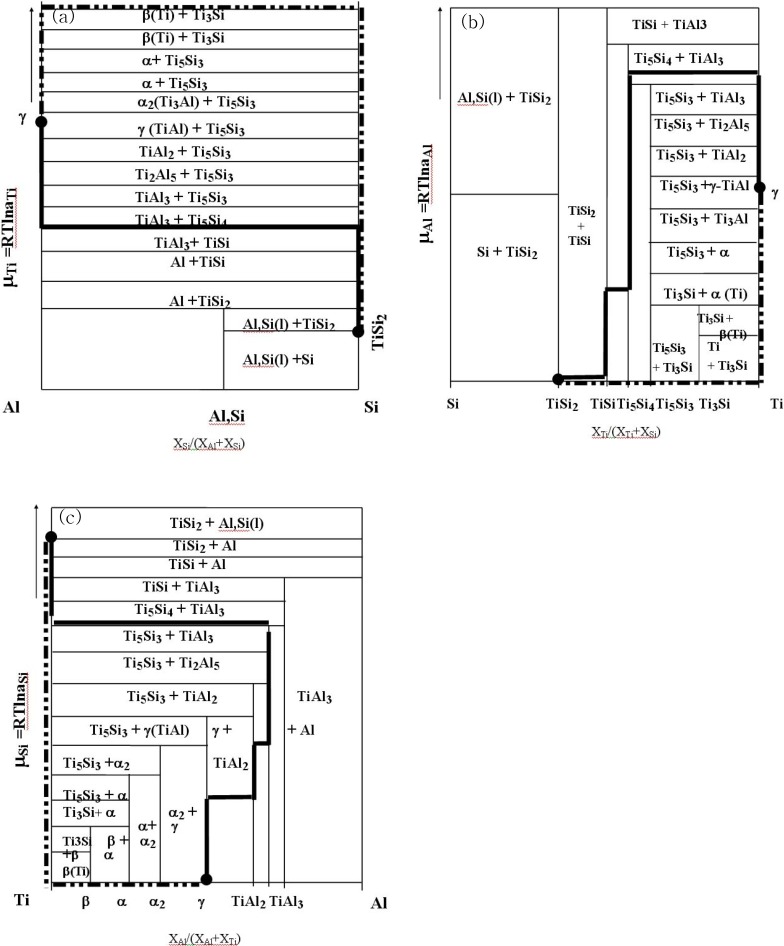
The stability diagram of Ti-Ai-Si ternary system with respect to (a) Ti (b)Al and (c) Si (Solid line: TiSi_2_*/*TiAl, dotted line: TiSi_2_*/*Ti*/*TiAl) [7].

Although the Ti_5_Si_3_ phase was not produced, the direction of the reaction pathway followed mass balance. The reaction pathway can be also understood by the reaction barrier effect. In the diffusion reaction of the Ti-Al system, the element Al has higher intrinsic diffusivity than the element Ti in the TiAl phase [57]. Based upon the binary results, the Al will move out the TiAl phase and yield titanium rich phases. However, the Al atoms become piled up in front of the Ti_5_Si_4_ phase due to the small solubility of Al in the titanium silicides for the current observations. It is considered that the Al contributed to the growth of the TiAl_3_ phase which is the Al rich phase. Actually, little aluminum is detected by EPMA in the Ti_5_Si_4_ region, resulting that the low solubility of the Al in the Ti_5_Si_4_ phase makes the diffusion of Al through the reaction zone difficult. Namely, the produced phase, Ti_5_Si_4_, which has no solubility of Al, may serve as a barrier to prevent Al diffusion to the TiSi_2_ side. It is interesting to note that the formation of a phase with low solubility for a ternary component results in a naturally built-in reaction barrier in the diffusion process. A similar effect has been reported for the reaction of the Ni_3_Al and SiC where the backward diffusion of the Al atoms occur [63], implying that the diffusion of components is affected by the solubility of the product phases. 

In order to investigate the chemical potential effect, the reaction pathway was indicated in the chemical potential diagram. Based upon the isothermal phase diagram, the free energy tangential diagram is initially constructed, as shown in Figure 18. It should be noted that the tangential plane is closely related to the isothermal phase diagram together with free energy values. Then, the stability diagrams with respect to the isothermal phase diagram (phase equilibria) were constructed based upon the estimated free energy as shown in Figure 18 (a), Figure 18 (b) and Figure 18 (c) with respect to Ti, Al and Si, respectively. The reaction pathway was marked in the stability diagram in order to visualize the developed diffusion pathway and the chemical potential variation. The solid lines indicate the reaction pathway of the TiSi_2_/TiAl, and the dotted lines mark the reaction pathway of the TiSi_2_*/*Ti*/*TiAl. In the reaction of the TiSi_2_*/*TiAl, the diffusion pathway shows that contrary to the elements Ti and Si, the chemical potential of Al was increased in the TiAl_3_ phase region. However, in the reaction of the TiAl*/*Ti*/*TiSi_2_, the chemical potential of Ti showed maximum in the Ti rich region. As discussed in the previously, from the thermodynamic point of view, the direction of a diffusion reaction is expected to occur with a lowering of the chemical potential. However, a reduction in chemical potential of each element is not mandated in order to reduce the overall free energy [9]. Therefore, it is clear that the chemical potential values of one component can be increased during a multiphase interdiffusion process in order to satisfy the mass balance requirement. Since the investigation of a chemical potential characterizes the diffusion pathway in a given system, the application of the diagram on the diffusion pathway and/or materials design is useful [62]. However, it should be mentioned that the current analyses works for three component systems, and the main point of the chemical potential diagram is based upon the thermodynamic estimation (local equilibrium), implying that this investigation provides excellent reference for estimating diffusion pathway in a semi-empirical manner. 

## 9. Summary

The identification of interface reactions in composite systems is critically important, since a proper estimation of the interface reactions can control the overall properties of these systems. Furthermore, the basic identification of the interface reactions should be used for optimizing and controlling diffusion reactions. In this work, the critical tools for analyzing the diffusion reactions and managing the basic data has been practiced for selected SiC/Metal and Ti-Al-Si ternary systems. For example, a practice of diffusion coupling experiments can give a lot of information for successfully tailored materials selections, when a materials system undergoes interdiffusion reactions. Furthermore, the basic information can be used for controlling diffusion pathway (product phases), interdiffusion parameters, and morphology controls, considering that the interface morphology is also one critical factor for governing the overall properties of composite systems together with kinetic parameters. Also, constructed chemical potential diagrams based upon thermodynamic and kinetic background can give a critical guidance for tailoring reaction pathways. Considering that the direction of diffusion pathway is mainly affected by the mass balance effect and that is controllable, one can optimize initial reactants in order to produce stable materials combinations. It appears that the attaining of the objective product phase by controlling the initial diffusion pathway is an excellent example showing the effectiveness of the application of the effective strategy in the composite interface design. 
